# Supplementary material for: Multilayer relaxor ferroelectric polymer stacks as data transmitter for real-time and programmable infrared information encryption
Source: Nat Commun. 2025 Nov 25;16:10448. doi: 10.1038/s41467-025-65419-2 (PMC12647881; doi:10.1038/s41467-025-65419-2)
Supplement: Supplementary file 1 — Supplementary Information [file 41467_2025_65419_MOESM1_ESM.pdf]

Supplementary Information

Multilayer Relaxor Ferroelectric Polymer Stacks as data transmitter for Real-time and Programmable Infrared Information Encryption

Yingke Zhu[1, †], Jianghan Wu[1, †], Yang Luo[1], Kede Liu[1], Hyeonji Hong[1], Yuxuan Guo[1], Yuan Meng[1], Meng Gao[1], Hanxiang Wu[1], Jiacheng Fan[1], Yingjie Du[1], Ping He[1], Qibing Pei[1*]

[1]Department of Materials Science and Engineering, University of California, Los Angeles, 420 Westwood Plaza, Los Angeles, California 90095, United States

*Corresponding author. Email: qpei@seas.ucla.edu

[†]These authors contributed equally: Yingke Zhu, Jianghan Wu.

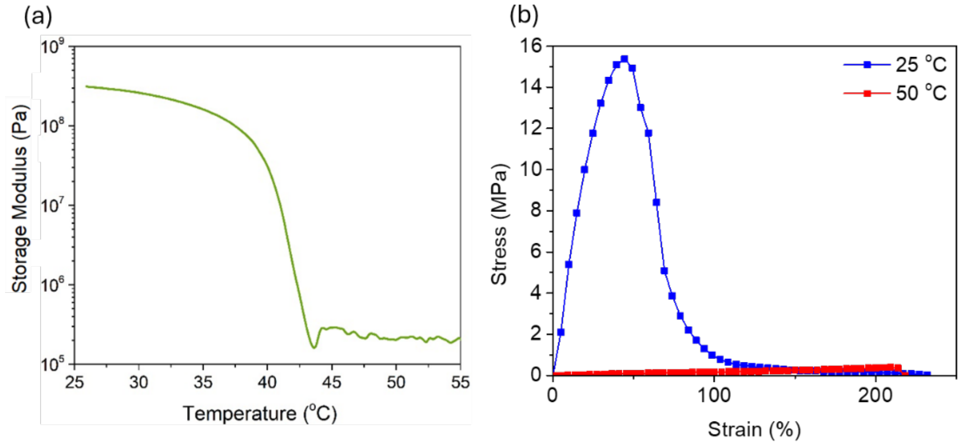

Supplementary Fig. 1 Mechanical properties of BAP: (a) Temperature-modulus curves of BAP. (b) Stress-strain curve of BAP at several different temperatures)

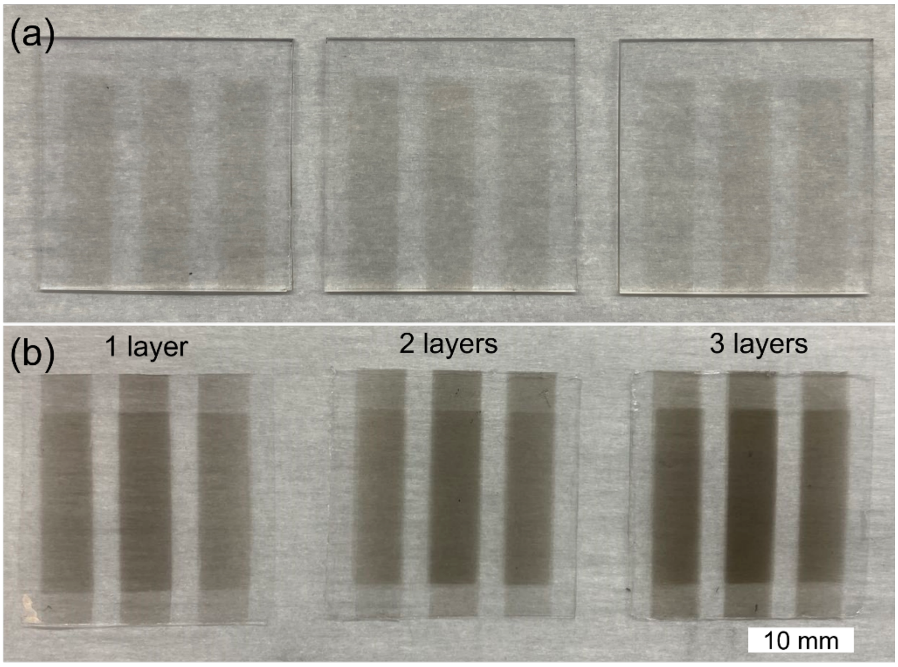

Supplementary Fig. 2 Optical images: (a) terpolymer with patterned CNT electrode on glass. (b)1 layer, 2 layers, and 3 layers of P(VDF-TrFE-CFE).

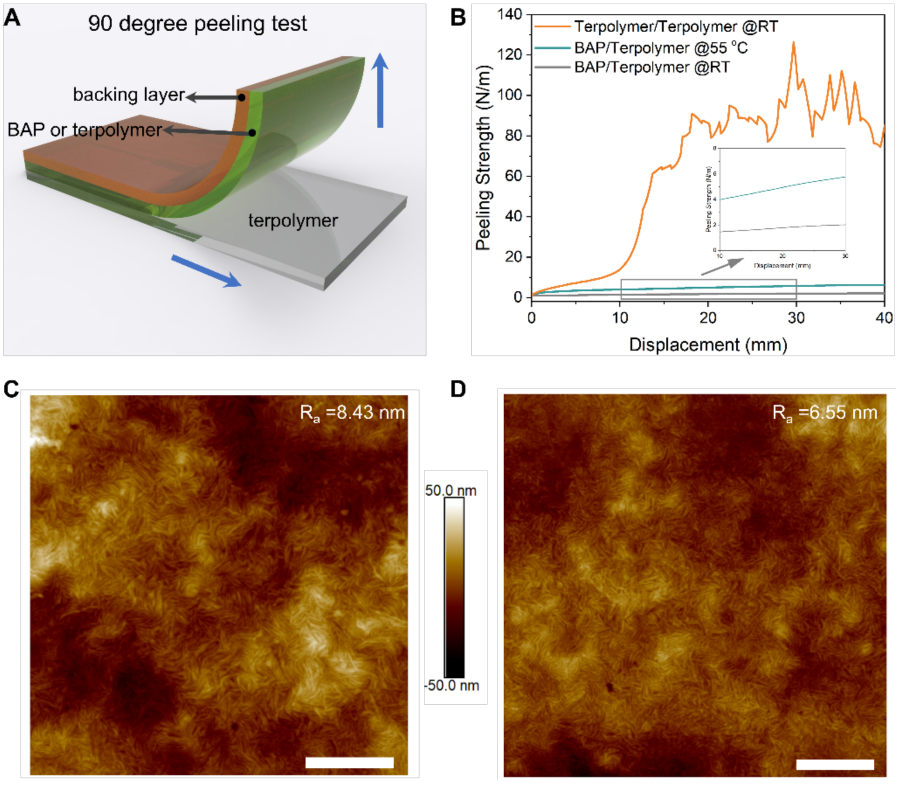

Supplementary Fig. 3 Peeling strength and surface morphology: (A) Schematic illustration of 90° peeling test. (B) Peel strength between different layers. Surface morphology of terpolymer layer (C) before and (D) after contact with BAP (scale bars are 1 μm).

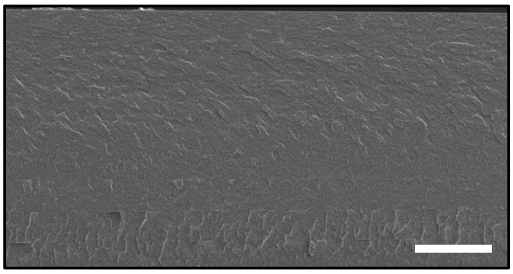

Supplementary Fig. 4 Cross-sectional SEM image of the 8-layer stack. The scale bar represents 50 μm.

	9320 P	9320 S
Detector Array	UFPA (VOx)	
Pixel Pitch	17 μm	
FOV	Lens dependent	
Measurement Distance	Lens dependent	
Pixel Resolution	320 x 240	
Spectral Band	7 μm to 14 μm	
Thermal Sensitivity (NETD)	< (20 mK) 0.02 °C at 30 °C (86 °F)	
Frame Rate	60 Hz	9 Hz
Dynamic Range	14-bit	
Temperature Range	25 °C to 45 °C (77°F to 113 °F)	
Operation Range	0 °C to 50 °C (32 °F to 122 °F)	
Storage Range	-40 °C to 80°C (-40 °F to 176 °F)	
Humidity	5% to 95% non-condensing	
Accuracy	± 0.2 °C (0.36 °F)	
Pixel Operability	> 99 %	
Shock/Vibration	70 G/4.3 G	
Dimensions (without lens)	37 mm x 37 mm x 41.5 mm (L x W x D ± 0.5 mm) (1.46” x 1.46” x 1.63” (L x W x H ± 0.02”))	
Power	5 V DC 500 mA, < 1.1 W	
Weight (without lens)	< 47 g (1.66 oz)	
Interface	USB Mini-B	
Video	Raw	
Emissivity Correction	0.01 to 1.0	
IP Rating	IP 54	
Shutter	Built-in shutter	

Supplementary Fig. 5 Camera parameters. Technical Specifications of IR Camera 9320P.

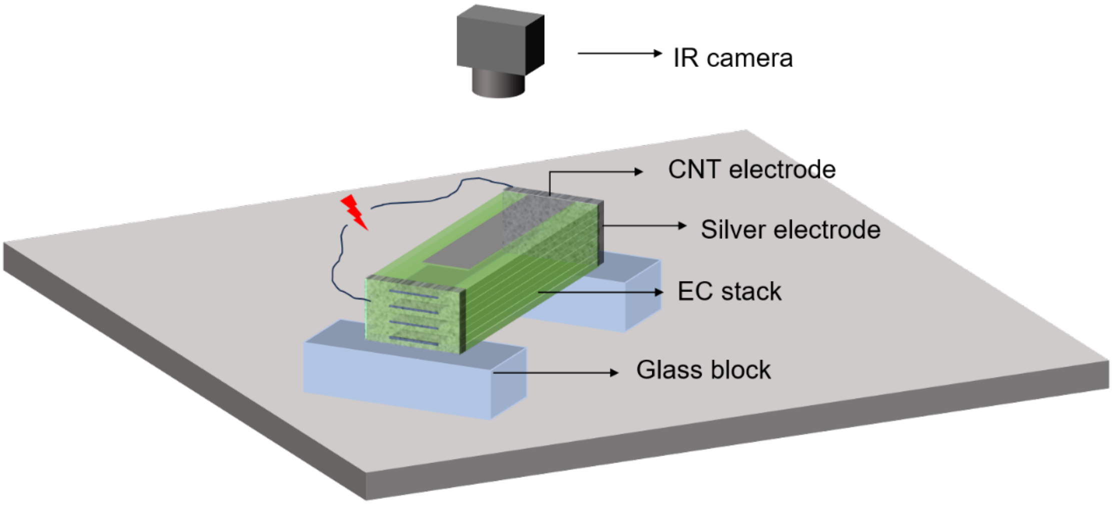

Supplementary Fig. 6 Electrocaloric test setup. Schematic illustration of ECE measurement setup.

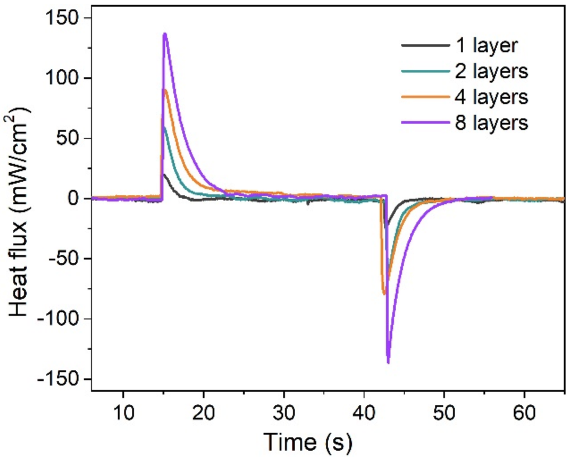

Supplementary Fig. 7 Heat flux results. Comparison of heat flux of different layers of stacks.

Supplementary Table 1 Measured Heat released and absorbed to the external heat sink.

Number of layers	Heat released (J/m^2)	Heat absorbed (J/m^2)
1	284	287
2	1093	1085
4	2629	1926
8	3921	2785

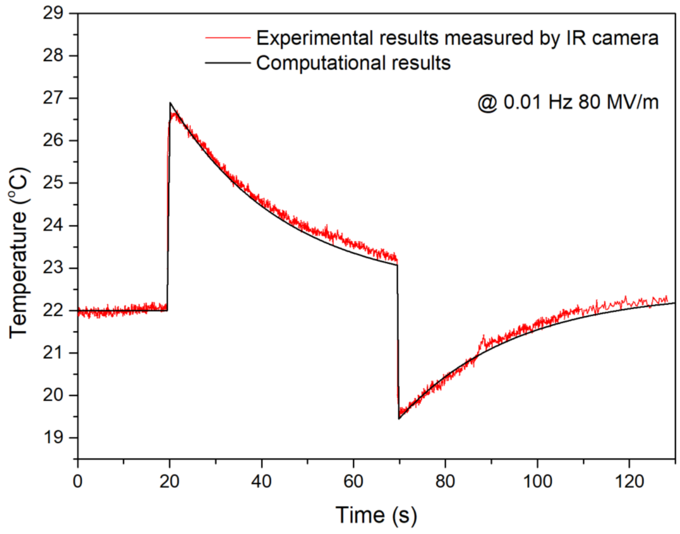

Supplementary Fig. 8 Comparison of experimental and computational results. Temperature profile of 8-layer EC stack, where the dots are recorded with IR camera and the solid lines are fitted data using Supplementary Equation (2).

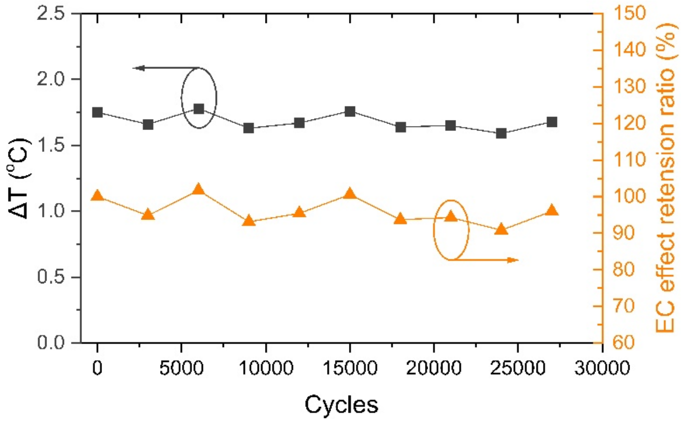

Supplementary Fig. 9 Cycling stability. Cyclic performance of 8-layer stack under 60 MV/m.

A	• —	N	— •	1	• — — — —
B	— • • •	O	— — —	2	• • — — —
C	— • — •	P	• — — •	3	• • • — —
D	— • •	Q	— — • —	4	• • • • —
E	•	R	• — •	5	• • • • •
F	• • — •	S	• • •	6	— • • • •
G	— — •	T	—	7	— — • • •
H	• • • •	U	• • —	8	— — — • •
I	• •	V	• • • —	9	— — — — •
J	• — — —	W	• — —	0	— — — — —
K	— • —	X	— • • —		
L	• — • •	Y	— • — —		
M	— —	Z	— — • •		

Supplementary Fig. 10 Morce code. International Morse code.

Supplementary Note 1

A simple model is developed to simulate the performance of one-layer and multilayer EC stack. For a one-layer film with a thickness of 50 μm, the Biot number is less than 0.1. It can be treated as a lumped heat capacity system.[1] The spatial temperature distribution within the EC film is ignored in the fitting procedure. For a multilayer stack, the EC temperature change of each layer is fast, simultaneous, and considered adiabatic, so the temperature distribution across eight layers is neglected. Therefore, the temperature decay after adiabatic temperature change is given by

$$hA(T(t) - T_R) = -cm\frac{dT(t)}{dt} \qquad (1)$$

from which we obtained the temperature profile

$$T(t) = T_R + \Delta T \exp\left(-\frac{hA}{cm}\,t\right) \qquad (2)$$

where h is the convective heat transfer coefficient, c is the specific heat of the stack, T_R is the ambient temperature, ΔT is the temperature difference between the object and the heat sink right after the adiabatic temperature change, m is the total mass of the stack, and t is the duration of temperature decay.[2] The thermal time constant $\tau = \frac{cm}{hA}$ represents the rate of temperature change in the heat transfer procedure, and τ was obtained from the measured temperature profile where the electric field is constant.[3] The computational temperature profile based on Supplementary Equation (2) is plotted

in Figure S8. The measured experimental data fits well with computational results.
In addition, by utilizing the high-voltage power supply (stepping up a 3.7 V DC input to 4 kV) build in our previous work[4], our stack can be driven by a 3.7 V DC. HV optocouplers (model OPTO-150, HVM Technology; turn-on/turn-off time = 2 μs) were used for switching in the high-voltage power supply. The voltage conversion speed is mainly determined by the power capacity of the optocouplers and the capacitance of the stack.

References

1. Gong J, McGaughey AJH. Device-level thermodynamic model for an electrocaloric cooler. *International Journal of Energy Research* **44**, 5343-5359 (2020).
2. Meng Y*, et al.* A cascade electrocaloric cooling device for large temperature lift. *Nat Energy* **5**, 996-1002 (2020).
3. Guo D*, et al.* Electrocaloric characterization of a poly (vinylidene fluoride-trifluoroethylene-chlorofluoroethylene) terpolymer by infrared imaging. *Applied Physics Letters* **105**, (2014).
4. Guo Y*, et al.* Haptic artificial muscle skin for extended reality. *Sci Adv* **10**, eadr1765 (2024).
