# Supplementary material for: Multilayer relaxor ferroelectric polymer stacks as data transmitter for real-time and programmable infrared information encryption
Source: Nat Commun. 2025 Nov 25;16:10448. doi: 10.1038/s41467-025-65419-2 (PMC12647881; doi:10.1038/s41467-025-65419-2)
Supplement: Supplementary file 2 — Description of Additional Supplementary Files [file 41467_2025_65419_MOESM2_ESM.pdf]

Description of Additional Supplementary Files

File name: Supplementary Movie 1
Description: Electrocaloric effect of 8 layers stack under 80 MV/m and 0.1 Hz.

File name: Supplementary Movie 2
Description: Encrypted temperature signals from “UCLA” captured by IR camera.
